# Human leukocyte antigen (HLA)-binding epitopes dataset for the newly identified T-cell antigens of *Mycobacterium immunogenum*

**DOI:** 10.1016/j.dib.2016.06.045

**Published:** 2016-07-05

**Authors:** Harish Chandra, Jagjit S. Yadav

**Affiliations:** Microbial Pathogenesis and Toxicogenomics Laboratory, Department of Environmental Health, University of Cincinnati College of Medicine, Cincinnati, OH 45267-0056, United States of America

**Keywords:** Immunoinformatics, T-cell antigen, T-cell epitopes, HLA-I, HLA-II, *Mycobacterium immunogenum*, *Mycobacterium tuberculosis*, Hypersensitivity pneumonitis

## Abstract

The dataset described herein is related to our article entitled “T-cell antigens of *Mycobacterium immunogenum* (MI)*,* an etiological agent of occupational hypersensitivity pneumonitis’’ (Chandra and Yadav, 2016) [1]. The data include *in silico*-predicted T-cell epitopes of the T-cell antigens AgA and AgD of MI predicted to bind to HLA-I or HLA-II alleles. Data on two reference T-cell antigens ESAT-6 and CFP-10 of *Mycobacterium tuberculosis* H37Rv are included for comparison. The data for each antigen include the predicted epitope׳s amino acid sequence, its first amino acid position, and its ability to bind HLA-I or HLA-II allele(s).

**Specifications Table**

**Table:** 

Subject area	Immunology
More specific subject area	Immunoinformatics
Type of data	Table
How data was acquired	*In silico* analysis of the T-cell antigens for epitopes
Data format	Analyzed
Experimental factors	The antigens were originally identified by 2D-immunoproteomics
Experimental features	T-cell reactive antigens were subjected to *in silico* analysis for HLA-I and HLA-II binding epitopes
Data source location	University of Cincinnati, College of Medicine, Cincinnati, OH, USA
Data accessibility	All relevant data provided in this article

**Value of the data**

• Occupational hypersensitivity pneumonitis (HP) is a T-cell-mediated disease caused by exposure to mycobacterial antigens via machining fluids contaminated with *Mycobacterium immunogenum* (MI). Currently there is no data on epitopes of this pathogen.
• The current data set relates to the first report on epitope prediction for the first set of T-cell antigens of this HP etiological agent.
• Knowledge of the T-cell epitopes which bind to HLA-I and HLA-II alleles in human population is significant as it can provide the needed information to develop further diagnostic tools and therapeutic or vaccine targets. HP is still a difficult-to-diagnose occupational lung disease due to the lack of such data.

## 1 Data

The dataset presented in this article is about epitopes of newly identified T-cell antigens of *Mycobacterium immunogenum* (MI) that causes HP [1], [2], [3]. The data show predicted HLA-I alleles for T-cell epitopes of AgA and AgD of MI (Tables 1 and 2) and ESAT-6 and CFP-10 of *M. tuberculosis* H37Rv (Tables 5 and 6), respectively. The corresponding data for predicted HLA-II alleles are presented in Tables 3 and 4 (MI antigens) and Tables 7 and 8 (reference antigens of *M. tuberculosis*), respectively.

## 2 Experimental design, materials and methods

The data in this article focus on two recombinant MI antigen proteins, AgA and AgD, selected based on the *in vitro* T-cell reactivity [1] from a pool of 33 seroreactive proteins of *M. immunogenum originally* identified in our previous 2D- immunoproteomics work [4], [5] . The selected antigens AgA and AgD were analyzed here for epitopes using *in silico* immunoinformatic approach. Amino acid sequences of the antigens were used for the *in silico* analysis. For comparison, two well-recognized immunodominant antigens ESAT-6 and CFP-10 of the tuberculosis agent *M*. *tuberculosis* were analyzed in parallel. The epitopes were predicted using ProPred-I and ProPred platforms that are meant to predict promiscuous binding regions for 47 and 51 different HLA class I and class II alleles, respectively [6].

## Footnotes

Appendix A Supplementary data associated with this article can be found in the online version at http://dx.doi.org/10.1016/j.dib.2016.06.045.

## References

[1] Chandra H., Yadav J.S. (2016). T-cell antigens of *Mycobacterium immunogenum,* an etiological agent of occupational hypersensitivity pneumonitis. Mol. Immunol..

[2] Rosenman K. (2015). Occupational diseases in individuals exposed to metal working fluids. Curr. Opin. Allergy. Clin. Immunol..

[3] Chandra H., Yadav E., Yadav J.S. (2013). Alveolar macrophage innate response to *Mycobacterium**immunogenum*, the etiological agent of hypersensitivity pneumonitis: role of JNK and p38 MAPK pathways. PLoS One.

[4] Gupta M.K., Subramanian V., Yadav J.S. (2009). Immunoproteomic identification of secretory and subcellular protein antigens and functional evaluation of the secretome fraction of *Mycobacterium**immunogenum*, a newly recognized species of the *Mycobacterium**chelonae- Mycobacterium**abscessus* group. J. Proteome Res..

[5] Chandra H., Lockey J., Yadav J.S. (2015). Novel antigens of *Mycobacterium**immunogenum* relevant in serodiagnosis of occupational hypersensitivity pneumonitis in machinists. Ann. Allergy Asthma Immunol..

[6] Singh H., Raghava G.P. (2001). ProPred: prediction of HLA-DR binding sites. Bioinformatics.

