# Supplementary material for: Human leukocyte antigen (HLA)-binding epitopes dataset for the newly identified T-cell antigens of Mycobacterium immunogenum
Source: Data Brief. 2016 Jul 5;8:1069–71. doi: 10.1016/j.dib.2016.06.045 (PMC4969084; doi:10.1016/j.dib.2016.06.045)
Supplement: Supplementary material [file mmc2.docx]

**Table 1:** **Predicted HLA-I alleles for T-cell epitopes of antigen AgA of *M. immunogenum***

| **Start**  **amino acid**  **position** | **Epitope** | **HLA-I allele(s)** |
| --- | --- | --- |
| 18 | VDHDQADAA | HLA-B*37:01,HLA-B40, HLA-B61,MHC-Kk |
| 21 | DQADAARQA | HLA-B*62 |
| 54 | WNARAAGHD | HLA-B*54:01 |
| 98 | LVKHPAHGL | HLA-B*7,HLA-B*8,MHC-Db revised |
| 100 | KHPAHGLTL | HLA-B*38:01,HLA-B*39:01,HLA-B*07:02,MHC-Db,MHC-Kb,MHC-Ld |
| 103 | AHGLTLVSL | HLA-B*38:01,HLA-B*39:01 |
| 105 | GLTLVSLDR | HLA-A3,HLA-A*31:01,HLA-A*33:02,HLA-A68.1,HLA-A20 Cattle |
| 112 | DRAVLEEVL | HLA-B14,HLA-B*39:02,HLA-Cw*06:02,HLA-Cw*07:02 |
| 145 | RGRIKQMLL | HLA-A24,HLA-B*35:01,HLA-B*37:01,HLA-B60,HLA-B7,HLA-B*07:02,HLA-B8,MHC-Dd |
| 154 | KIGWPAEDL | HLA-A*02:01,HLA-A*02:05 |
| 167 | DGEAHPITL | HLA-A1,HLA-B*51:01,HLA-B60,MHC-Dd |
| 185 | VDHDQADAA | MHC-Kk |
| 186 | QQMAADSFW | HLA-B*52:01,HLA-B62 |
| 195 | AGGSGVVVL | HLA-A2.1,HLA-B*51:03,HLA-B*52:01,HLA-Cw*04:01,MHC-Dd,MHC-Kd |
| 203 | LPCGAGKTL | HLA-B*35:01,HLA-B*38:01,HLA-B*51:01,HLA-B*51:02,HLA-B*53:01,HLA-B*54:01,HLA-B*51,HLA-B7,HLA-Cw*04:01,MHC-Ld |
| 206 | GAGKTLVGA | HLA-B*58:01, HLA-B*61 |
| 209 | KTLVGAAAM | HLA-A2,HLA-B*58:01,HLA-B*07:02,MHC-Db |
| 211 | LVGAAAMAK | HLA-A*11:01,HLA-A3,HLA-A68.1 |
| 217 | MAKAQATTL | HLA-B*35:01,HLA-B*51:01,HLA-B*51:02,HLA-B*51:03,HLA-B*58:01,HLA-B8,MHC-Db revised |
| 219 | KAQATTLIL | HLA-A24,HLA-B*35:01,HLA-B*51:02,HLA-B*51:03,HLA-B*52:01,HLA-B*58:01,MHC-Kb,MHC-Ld |
| 239 | RELLARTSL | HLA-A*02:05,HLA-B*37:01,HLA-B40,HLA-B*44:03,HLA-B60, HLA-B61,HLA-B*07:02,MHC-Kk |
| 303 | LPAPVFRMT | HLA-B*52:01, HLA-B*53:01,HLA-B*51,HLA-Cw*07:02,MHC-Ld |
| 362 | ECIEVRVTL | HLA-A24,HLA-B14,HLA-B*39:02,HLA-B40,HLA-B60,HLA-B8,HLA-Cw*03:01,MHC-Db,MHC-Kd |
| 405 | ARHPGAPTL | HLA-B14,HLA-B*27:02,HLA-Cw*03:01 |
| 417 | AYLDQLDEL | HLA-A24,HLA-Cw*03:01,HLA-Cw*04:01,HLA-Cw*07:02,MHC-Db,MHC-Kd |
| 421 | QLDELGTEL | HLA-A2, HLA-A20 Cattle,HLA-A2.1,HLA-B*38:01,HLA-B*39:02,HLA-B62,HLA-Cw*04:01,HLA-Cw*06:02,MHC-Kd |
| 438 | TKNAEREAL | HLA-A20 Cattle,HLA-B*39:02,MHC-Db revised |
| 450 | FRRGEIGTL | HLA-B*27:02, HLA-B*27:05,HLA-B*53:01,HLA-B*54:01,HLA-B*51 |
| 462 | KVANFSIDL | HLA-A2,HLA-A*02:01,HLA-A*02:05,HLA-A3,HLA-A68.1,HLA-A2.1,HLA-B7,HLA-Cw*06:02,HLA-Cw*07:02,MHC-Db revised |
| 467 | SIDLPEASV | HLA-A2,HLA-A*02:01 |
| 485 | SRQEEAQRL | HLA-B*27:02,HLA-B*27:05,HLA-B*39:01,MHC-Kb |
| 489 | EAQRLGRLL | HLA-B*37:01,HLA-B*51:01,HLA-B*51:02,HLA-B*51:03,HLA-Cw*06:02,MHC-Kb |
| 492 | RLGRLLRPK | HLA-A3,HLA-A68.1,HLA-A20 Cattle |
| 536 | YRITDADD | HLA-A2.1,HLA-B14,HLA-B*27:02,HLA-B*27:05,HLA-B*39:01,HLA-B*53:01,HLA-B*54:01,HLA-B*51,HLA-Cw*03:01 |

**Table 2:** **Predicted HLA-I alleles for T-cell epitopes of antigen AgD of *M. immunogenum***

| **Start amino acid position** | **Epitope** | **HLA-I allele(s)** |
| --- | --- | --- |
| 20 | HVDHGKTTL | HLA A*0205,HLA A68.1,HLA B*3801,HLA B*0702 |
| 22 | DHGKTTLTA | HLA B*3801 |
| 28 | LTAAITKVL | HLA A68.1,HLA B*3902,HLA B*5201,HLA B*5801,HLA B60,HLA Cw*0602,MHC Kb |
| 35 | VLHDKYPDL | HLA A2,HLA A*0201,HLA A*0205,HLA A20 Cattle,HLA A2.1,HLA B*3901,HLA B62,HLA B8,MHC Dd,MHC Kb, |
| 39 | KYPDLNEAS | HLA A24,HLA Cw*0702,MHC Dd |
| 79 | YAHVDAPGH | HLA B*5401 |
| 93 | NMITGAAQM | HLA A2,HLA A*0201,HLA A20 Cattle,MHC Db,MHC Db revised,MHC Kb |
| 101 | MDGAILVVA | HLA B40 |
| 102 | DGAILVVAA | HLA B*4403 |
| 115 | MPQTREHVL | HLA-B14,HLA-B*3501,HLA-B*5101,HLA-B*5102,HLA-B*5103,HLA-B*5201,HLA-B*5301,HLA-B*51,HLA-B7,HLA-B*0702,HLA-B8,HLA-Cw*0401,MHC-Dd,MHC-Ld |
| 126 | RQVGVPYIL | HLA A2,HLA A*0205,HLA A24,HLA B*2702,HLA B*2705,HLA B*3902,HLA B*5102,HLA B*5201,HLA B62,HLA B*0702,HLA Cw*0301,MHC Db revised,MHC Kd |
| 140 | SDMVDDEEL | HLA B*3701,HLA B40,HLA B*4403 |
| 141 | DMVDDEELL | HLA A2,HLA A*0201,HLA A24,HLA A*3302,HLA A20 Cattle,HLA A2.1,HLA B14,MHC Db,MHC Db revised,MHC Kd |
| 165 | FDGDNAPVV | HLA B*3701,HLA B*5201,HLA B*5301,HLA B*5401,HLA B*51, HLA B61,MHC Db,MHCKk |
| 167 | GDNAPVVRV | HLA B*3701, HLA B61,MHC Kk |
| 170 | APVVRVSAL | HLA B14,HLA B*3501,HLA B*3901,HLA B40,HLA B*5101,HLA B*5102,HLA B*5103,HLA B*5301,HLA B*5401,HLA B*51,HLA B7,HLA B*0702,HLA B8,HLA Cw*0301,HLA Cw*0401 |
| 173 | VRVSALKAL | HLA B14,HLA B*2702,HLA B*2705,HLA B*3901,HLA Cw*0301,HLA Cw*0702,MHC Db |
| 172 | VVRVSALKA | HLA A68.1 |
| 183 | GDAEWGKTV | HLA A2.1,HLA B61,MHC Kk |
| 186 | EWGKTVADL | MHC Kd |
| 194 | LMDAVDESI | HLA A*0201,HLA A20 Cattle,HLA A2.1,HLA B*5101,HLA B*5103,MHC Kk |
| 206 | VRETEKPFL | HLA B*2702,HLA B*2705,HLA B*3901 |
| 261 | GVEMFRKLL | HLA A*0205,HLA A68.1,HLA B*3801,HLA B*3902,HLA B7,HLA Cw*0602, |
| 271 | GQAGDNVGL | MHC Db revised |
| 272 | GQAGDNVGL | HLA B*2702,HLA B*2705,HLA B*3902,HLA B62 |
| 339 | TDVTGVVTL | HLA B*3701,HLA B40,HLA B*4403,HLA B60,HLACw*0301 |
| 358 | DNTDISVKL | HLA A24,HLA B*3801,HLA B60,HLA Cw*0602,MHC Kb |
| 368 | QPVAMDEGL | HLA B*3501,HLA B*5101,HLA B*5102,HLA B*5103,HLA B*5301,HLA B*5401,HLA B*51,HLA B7,HLA Cw*0401,HLA Cw*0602,HLA Cw*0702,MHC Kd,MHC Ld |
| 370 | VAMDEGLRF | HLA B*3501,HLA B*5801,HLA B62,MHC Ld |

**Table 3:** **Predicted HLA-II alleles for T-cell epitopes of antigen AgA of *M. immunogenum***

| **Start**  **amino acid position** | **Epitope** | **HLA-II allele(s)** |
| --- | --- | --- |
| 45 | YRITPLALW | HLA-DRB1*13:07,HLA-DRB1*13:21 |
| 50 | LALWNARAA | HLA-DRB1*04:02 |
| 53 | WNARAAGHD | HLA-DRB1*08:01 |
| 65 | VVDALVTFS | HLA-DRB1*04:04,HLA-DRB1*04:23 |
| 73 | VQSDKTVLL | HLA-DRB1*11:07 |
| 81 | LLVDIVDTM | HLA-DRB1*03:01, HLA-DRB1*03:06,HLA-DRB1*03:07,HLA-DRB1*03:08,HLA-DRB1*03:09,HLA-DRB1*03:11,HLA-DRB1*011:07 |
| 89 | MARYGRLQL | HLA-DRB1*15:01,HLA-DRB1*15:02,HLA-DRB1*15:06 |
| 97 | LVKHPAHGL | HLA-DRB1*01:01,HLA-DRB1*01:02,HLA-DRB1*07:01,HLA-DRB1*07:03,HLA-DRB1*013:04 |
| 107 | LVSLDRAVL | HLA-DRB1*01:02,HLA-DRB1*11:04,HLA-DRB1*11:06,HLA-DRB1*11:28,HLA-DRB1*13:05,HLA-DRB1*13:07,HLA-DRB1*13:11,HLA-DRB1*13:21 |
| 119 | LRHKKIAPM | HLA-DRB1*08:01,HLA-DRB1*08:02,HLA-DRB1*08:04,HLA-DRB1*08:06,HLA-DRB1*08:13,HLA-DRB1*08:17,HLA-DRB1*11:02,HLA-DRB1*11:20,HLA-DRB1*11:21,HLA-DRB1*13:01,HLA-DRB1*13:04,HLA-DRB1*13:02,HLA-DRB1*13:22,HLA-DRB1*13:27,HLA-DRB1*13:28 |
| 199 | VVVLPCGAG | HLA-DRB1*01:02 |
| 210 | LVGAAAMAK | HLA-DRB1*11:04,HLA-DRB1*11:06,HLA-DRB1*13:11,HLA-DRB5*01:01,HLA-DRB5*01:05 |
| 224 | LILVTNTVA | HLA-DRB1*04:04,HLA-DRB1*04:05,HLA-DRB1*04:10,HLA-DRB1*04:23,HLA-DRB1*04:08 |
| 280 | YRHLELFDS | HLA-DRB1*01:01,HLA-DRB1*03:05,HLA-DRB1*04:05,HLA-DRB1*04:08,HLA-DRB1*08:02,HLA-DRB1*08:13,HLA-DRB1*11:01,HLA-DRB1*11:02,HLA-DRB1*11:14,HLA-DRB1*11:20,HLA-DRB1*11:21,HLA-DRB1*11:28,HLA-DRB1*13:02,HLA-DRB1*13:05,HLA-DRB1*13:07,HLA-DRB1*13:21,HLA-DRB1*13:22,HLA-DRB1*13:23,HLA-DRB1*15:02,HLA-DRB5*01:01,HLA-DRB5*01:05 |
| 269 | YQVITRRTK | HLA-DRB1*11:01,HLA-DRB1*11:14,HLA-DRB1*11:28,HLA-DRB1*13:05,HLA-DRB1*13:07,HLA-DRB1*13:23,HLA-DRB5*01:01,HLA-DRB5*01:05 |
| 271 | VITRRTKGE | HLA-DRB1*08:01,HLA-DRB1*08:06 |
| 294 | IVYDEVHLL | HLA-DRB1*03:01,HLA-DRB1*03:05,HLA-DRB1*03:06,HLA-DRB1*03:07,HLA-DRB1*03:08,HLA-DRB1*03:11,HLA-DRB1*04:01,HLA-DRB1*04:21,HLA-DRB1*04:26,HLA-DRB1*011:07 |
| 299 | VHLLPAPVF | HLA-DRB1*01:01,HLA-DRB1*01:02 |
| 301 | LLPAPVFRM | HLA-DRB1*13:01,HLA-DRB1*13:02,HLA-DRB1*13:04,HLA-DRB1*13:27,HLA-DRB1*13:28 |
| 306 | VFRMTADLQ | HLA-DRB1*04:10 |
| 307 | FRMTADLQS | HLA-DRB1*04:01,HLA-DRB1*04:08,HLA-DRB1*04:21,HLA-DRB1*04:26,HLA-DRB1*11:01,HLA-DRB1*11:28,HLA-DRB1*13:05,HLA-DRB1*15:02 |
| 313 | LQSRRRLGL | HLA-DRB1*08:04,HLA-DRB1*08:06,HLA-DRB1*08:17,HLA-DRB1*15:01,HLA-DRB1*15:06 |
| 319 | LGLTATLIR | HLA-DRB1*04:21 |
| 336 | FSLIGPKRY | HLA-DRB5*01:01,HLA-DRB5*01:05 |
| 363 | IEVRVTLTD | HLA-DRB1*08:17 |
| 365 | VRVTLTDNE | HLA-DRB1*04:10 |
| 386 | YKLCSTAHT | HLA-DRB1*04:05,HLA-DRB1*04:08 |
| 396 | INVVKSILA | HLA-DRB1*04:04,HLA-DRB1*04:23 |
| 412 | LVIGAYLDQ | HLA-DRB1*03:06,HLA-DRB1*03:07,HLA-DRB1*03:08,HLA-DRB1*03:11 |
| 457 | VQVSGTFGS | HLA-DRB1*03:01, HLA-DRB1*03:05,HLA-DRB1*04:01,HLA-DRB1*04:02,HLA-DRB1*04:04,HLA-DRB1*04:10,HLA-DRB1*04:23,HLA-DRB1*04:26,HLA-DRB1*011:07 |
| 458 | VVSKVANFS | HLA-DRB1*04:02, HLA-DRB1*08:02,HLA-DRB1*08:04,HLA-DRB1*08:13,HLA-DRB1*11:02,HLA-DRB1*11:14,HLA-DRB1*11:20,HLA-DRB1*11:21,HLA-DRB1*13:01,HLA-DRB1*13:02,HLA-DRB1*13:04,HLA-DRB1*13:22,HLA-DRB1*13:23,HLA-DRB1*13:28,HLA-DRB1*13:27 |
| 465 | FSIDLPEAS | HLA-DRB1*03:05,HLA-DRB1*03:06,HLA-DRB1*03:07,HLA-DRB1*03:08,HLA-DRB1*03:09,HLA-DRB1*03:11,HLA-DRB1*04:01,HLA-DRB1*04:26 |
| 476 | VQVSGTFGS | HLA-DRB1*03:01,HLA-DRB1*03:05,HLA-DRB1*04:01,HLA-DRB1*04:02,HLA-DRB1*04:04,HLA-DRB1*04:10,HLA-DRB1*04:23,HLA-DRB1*04:26,HLA-DRB1*11:07 |
| 492 | LGRLLRPKA | HLA-DRB1*11:04,HLA-DRB1*11:06,HLA-DRB1*13:11 |
| 506 | YFYSVVSRD | HLA-DRB1*04:05 |
| 510 | VVSRDTLDA | HLA-DRB1*04:02,HLA-DRB1*08:01,HLA-DRB1*08:02,HLA-DRB1*08:04,HLA-DRB1*08:06,HLA-DRB1*08:13,HLA-DRB1*08:17,HLA-DRB1*11:01,HLA-DRB1*11:02,HLA-DRB1*11:04,HLA-DRB1*11:06,HLA-DRB1*11:14,HLA-DRB1*11:21,HLA-DRB1*13:11,HLA-DRB1*13:22,HLA-DRB1*13:23,HLA-DRB1*15:01,HLA-DRB1*15:06 |
| 535 | YRITDADDL | HLA-DRB1*01:01,HLA-DRB1*07:01,HLA-DRB1*07:03 |

**Table 4:** **Predicted HLA-II alleles for T-cell epitopes of antigen AgD of *M. immunogenum***

| **Start**  **amino acid position** | **Epitope** | **HLA -II allele(s)** |
| --- | --- | --- |
| 12 | VNIGTIGHV | HLA-DRB1*11:01,HLA-DRB1*11:04,HLA-DRB1*11:06,HLA-DRB1*13:07,HLA-DRB1*13:11 |
| 64 | INISHVEYQ | HLA-DRB1*03:01,HLA-DRB1*03:06,HLA-DRB1*03:07,HLA-DRB1*03:08,HLA-DRB1*03:11 |
| 90 | IKNMITGAA | HLA-DRB1*04:05 |
| 105 | LVVAATDGP | HLA-DRB1*04:05,HLA-DRB1*04:10,HLA-DRB1*08:17,HLA-DRB1*13:21 |
| 121 | VLLARQVGV | HLA-DRB1*01:01,HLA-DRB1*04:01,HLA-DRB1*04:08,HLA-DRB1*04:21,HLA-DRB1*04:26,HLA-DRB1*07:01,HLA-DRB1*07:03 |
| 122 | LLARQVGVP | HLA-DRB1*08:01,HLA-DRB1*08:02,HLA-DRB1*08:13 |
| 131 | YILVALNK | HLA-DRB1*03:05,HLA-DRB1*03:06,HLA-DRB1*03:07,HLA-DRB1*03:08,HLA-DRB1*03:11,HLA-DRB1*04:01,HLA-DRB1*04:04,HLA-DRB1*04:21,HLA-DRB1*04:23,HLA-DRB1*04:26,HLA-DRB1*11:02,HLA-DRB1*11:07,HLA-DRB1*11:14,HLA-DRB1*11:21,HLA-DRB1*13:22,HLA-DRB1*13:23 |
| 133 | LVALNKSDM | HLA-DRB1* 01:02 |
| 150 | LVEMEVREL | HLA-DRB1*01:01,HLA-DRB1*01:02 |
| 153 | MEVRELLSS | HLA-DRB1*04:02 |
| 155 | VRELLSSQD | HLA-DRB1*04:02,HLA-DRB1*08:01,HLA-DRB1*08:02,HLA-DRB1*08:04,HLA-DRB1*08:06,HLA-DRB1*08:13,HLA-DRB1*08:17 |
| 171 | VVRVSALKA | HLA-DRB1*01:01,HLA-DRB1*01:02,HLA-DRB1*03:05,HLA-DRB1*03:07,HLA-DRB1*03:06,HLA-DRB1*03:08,HLA-DRB1*03:11,HLA-DRB1*04:02,HLA-DRB1*04:04,HLA-DRB1*04:08,HLA-DRB1*04:10,HLA-DRB1*04:23,HLA-DRB1*07:01,HLA-DRB1*07:03,HLA-DRB1*08:02,HLA-DRB1*08:04,HLA-DRB1*08:17,HLA-DRB1*11:01,HLA-DRB1*11:02,HLA-DRB1*11:04, HLA-DRB1*11:06,HLA-DRB1*11:07,HLA-DRB1*11:21,HLA-DRB1*11:28,HLA-DRB1*13:05,HLA-DRB1*13:07,HLA-DRB1*13:11,HLA-DRB1*13:21,HLA-DRB1*13:22,HLA-DRB1*15:01,HLA-DRB1*15:02,HLA-DRB1*15:06,HLA-DRB5*01:01,HLA-DRB5*01:05 |
| 219 | VFTITGRGT | HLA-DRB1*01:02,HLA-DRB1*04:02,HLA-DRB1*11:02,HLA-DRB1*11:14,HLA-DRB1*11:20,HLA-DRB1*11:21,HLA-DRB1*13:01,HLA-DRB1*13:02,HLA-DRB1*13:04,HLA-DRB1*13:22,HLA-DRB1*13:23,HLA-DRB1*13:27,HLA-DRB1*13:28,HLA-DRB5*01:01,HLA-DRB5*01:05 |
| 220 | FTITGRGTV | HLA-DRB1*11:07 |
| 228 | VVTGRVERG | HLA-DRB1*03:01,HLA-DRB1*15:01,HLA-DRB1*15:06 |
| 247 | VGIKDTTTK | HLA-DRB1*04:01,HLA-DRB1*04:21,HLA-DRB1*04:26 |
| 261 | VEMFRKLLD | HLA-DRB1*08:01,HLA-DRB1*08:04,HLA-DRB1*08:06,HLA-DRB1*08:17 |
| 264 | FRKLLDQGQ | HLA-DRB1*03:06,HLA-DRB1*03:07,HLA-DRB1*03:08,HLA-DRB1*03:11,HLA-DRB1*04:01,HLA-DRB1*04:26,HLA-DRB1*11:07 |
| 277 | VGLLVRGVK | HLA-DRB1*08:06,HLA-DRB1*13:01,HLA-DRB1*13:04,HLA-DRB1*13:27,HLA-DRB1*13:28 |
| 279 | LLVRGVKRE | HLA-DRB1*07:01,HLA-DRB1*07:03 |
| 280 | LVRGVKRED | HLA-DRB1*08:04 |
| 281 | VRGVKREDV | HLA-DRB1*04:21, |
| 284 | VKREDVERG | HLA-DRB1*04:01,HLA-DRB1*04:05,HLA-DRB1*04:08,HLA-DRB1*04:26 |
| 296 | VKPGTTTPH | HLA-DRB1*04:02,HLA-DRB1*04:04,HLA-DRB1*04:23,HLA-DRB1*08:13,HLA-DRB1*11:02,HLA-DRB1*11:21,HLA-DRB1*13:22 |
| 325 | FFNNYRPQ | HLA-DRB1*03:05, HLA-DRB1*03:09,HLA-DRB1*04:05,HLA-DRB1*04:08,HLA-DRB1*08:02,HLA-DRB1*08:13,HLA-DRB1*11:01,HLA-DRB1*11:14,HLA-DRB1*11:20,HLA-DRB1*13:02,HLA-DRB1*13:07,HLA-DRB1*13:23,HLA-DRB1*15:02,HLA-DRB5*01:01,HLA-DRB5*01:01 |
| 326 | FNNYRPQF | HLA-DRB1*08:01,HLA-DRB1*08:06,HLA-DRB1*08:17,HLA-DRB1*11:01,HLA-DRB1*11:04,HLA-DRB1*11:06,HLA-DRB1*11:20,HLA-DRB1*11:28,HLA-DRB1*13:01,HLA-DRB1*13:02,HLA-DRB1*13:04,HLA-DRB1*13:05,HLA-DRB1*13:11,HLA-DRB1*13:21,HLA-DRB1*13:27,HLA-DRB1*13:28,HLA-DRB1*15:01,HLA-DRB1*15:02,HLA-DRB1*15:06 |
| 329 | YRPQFYFRT | HLA-DRB1*01:01,HLA-DRB1*01:02,HLA-DRB1*03:05,HLA-DRB1*03:07,HLA-DRB1*03:06,HLA-DRB1*03:08,HLA-DRB1*03:11,HLA-DRB1*04:02,HLA-DRB1*04:04,HLA-DRB1*04:08,HLA-DRB1*04:10,HLA-DRB1*04:23,HLA-DRB1*07:01,HLA-DRB1*07:03,HLA-DRB1*08:02,HLA-DRB1*08:04,HLA-DRB1*08:17,HLA-DRB1*11:01,HLA-DRB1*11:02,HLA-DRB1*11:04,HLA-DRB1*11:06,HLA-DRB1*11:07,HLA-DRB1*11:21,HLA-DRB1*11:28,HLA-DRB1*13:05 |
| 335 | FRTTDVTGV | HLA-DRB1*01:01,HLA-DRB1*15:02 |
| 340 | VTGVVTLPE | HLA-DRB1*04:04,HLA-DRB1*04:05,HLA-DRB1*04:08, HLA-DRB1*04:10,HLA-DRB1*04:23,HLA-DRB1*08:02,HLA-DRB1*08:04,HLA-DRB1*11:01,HLA-DRB1*11:04,HLA-DRB1*11:06,HLA-DRB1*13:07,HLA-DRB1*13:11 |
| 354 | MPGDNTDIS | HLA-DRB1*03:05,HLA-DRB1*03:09,HLA-DRB1*11:20,HLA-DRB1*11:28,HLA-DRB1*13:02,HLA-DRB1*13:05 |
| 363 | VKLIQPVAM | HLA-DRB1*01:01,HLA-DRB1*03:01,HLA-DRB1*01:02,HLA-DRB1*03:09,HLA-DRB1*04:04,HLA-DRB1*04:10,HLA-DRB1*04:21,HLA-DRB1*04:23,HLA-DRB1*07:01,HLA-DRB1*07:03,HLA-DRB1*11:28,HLA-DRB1*13:05,HLA-DRB1*13:21 |
| 369 | VAMDEGLRF | HLA-DRB1*03:01,HLA-DRB1*03:06,HLA-DRB1*03:07,HLA-DRB1*03:08,HLA-DRB1*03:09,HLA-DRB1*03:11,HLA-DRB1*13:01,HLA-DRB1*13:27,HLA-DRB1*13:28 |
| 375 | LRFAIREGG | HLA-DRB1*03:01,HLA-DRB1*03:09,HLA-DRB1*08:01,HLA-DRB1*08:06,HLA-DRB1*08:13,HLA-DRB1*11:02,HLA-DRB1*11:04,HLA-DRB1*11:06,HLA-DRB1*11:07,HLA-DRB1*11:14,HLA-DRB1*11:20,HLA-DRB1*11:21,HLA-DRB1*11:28,HLA-DRB1*13:01,HLA-DRB1*13:02,HLA-DRB1*13:04,HLA-DRB1*13:05,HLA-DRB1*13:07,HLA-DRB1*13:11,HLA-DRB1*13:21,HLA-DRB1*13:22,HLA-DRB1*13:23,HLA-DRB1*13:27,HLA-DRB1*13:28 |

| **Start amino acid**  **position** | **Epitope** | **HLA- I allele(s)** |
| --- | --- | --- |
| 4 | QQWNFAGIE | HLA-B*27:05,HLA-B*52:01, |
| 6 | WNFAGIEAA | HLA-B40,HLA-B*53:01,HLA-B*54:01,HLA-B*51,HLA-B61, |
| 8 | FAGIEAAAS | HLA-B*51:01,HLA-B*51:02,HLA-B*51:03,HLA-B*54:01,HLA-B*51, |
| 20 | GNVTSIHSL | HLA-A24,HLA-A20 Cattle,HLA-A2.1,HLA-B14,HLA-B*27:05,HLA-B*39:01,HLA-B*39:02,HLA-B40,HLA-B*51:02,HLA-B*53:01,HLA-B60,HLA-B7,HLA-Cw*03:01,HLA-Cw*04:01,HLA-Cw*06:02,HLA-Cw*07:02,MHC-Db,MHC-Db revised,MHC-Kb,MHC-Kd,MHC-Ld |
| 28 | LLDEGKQSL | HLA-A2,HLA-A*02:01,HLA-A*02:05,HLA-A20 Cattle,HLA-A2.1,HLA-B*27:05,HLA-B*38:01,HLA-B*39:01,HLA-B*39:02,HLA-B*52:01,HLA-B*53:01,HLA-B*51,MHC-Kd |
| 31 | EGKQSLTKL | HLA-B14,HLA-B*35:01,HLA-B*51:01,HLA-B*51:02,HLA-B*51:03,HLA-B*52:01,HLA-B60,HLA-B7,HLA-B*07:02,HLA-B8,HLA-Cw*04:01,HLA-Cw*06:02,HLA-Cw*07:02,MHC-Db,MHC-Dd, |
| 32 | GKQSLTKLA | HLA-A2,HLA-A20 Cattle,HLA-B*53:01, |
| 33 | KQSLTKLAA | HLA-A*02:01,HLA-A*02:05,HLA-B*27:02,HLA-B*27:05,HLA-B*52:01,HLA-B62,HLA-B*07:02, |
| 34 | QSLTKLAAA | HLA-B*54:01,HLA-B*58:01,HLA-B*07:02,MHC-Ld |
| 51 | YQGVQQKWD | HLA-B*54:01,HLA-B*51,MHC-Db revised, |
| 57 | KWDATATEL | HLA-A24,HLA-A2.1,HLA-B*38:01,HLA-B*39:01,HLA-B*39:02,HLA-B*07:02,HLA-Cw*04:01,MHC-Dd,MHC-Kd |
| 59 | DATATELNN | HLA-B*51:01,HLA-B*51:03, |
| 61 | TATELNNAL | HLA-A*02:01,HLA-A*02:05,HLA-A24,HLA-B*35:01,HLA-B*38:01,HLA-B*39:01,HLA-B*39:02,HLA-B*51:01,HLA-B*51:02,HLA-B*51:03,HLA-B*58:01,HLA-B60,HLA-B7,HLA-B8,HLA-Cw*03:01,HLA-Cw*06:02,HLA-Cw*07:02,MHC-Db,MHC-Db revised,MHC-Dd,MHC-Kb,MHC-Kd,MHC-Ld |
| 64 | ELNNALQNL | HLA-A2,HLA-A*02:01,HLA-A*02:05,HLA-A24,HLA-A20 Cattle,HLA-A2.1,HLA-B14,HLA-B*38:01,HLA-B62,HLA-B7,HLA-B8,HLA-Cw*03:01,HLA-Cw*04:01,HLA-Cw*06:02,HLA-Cw*07:02,MHC-Db,MHC-Dd,MHC-Kb,MHC-Ld |

**Table 5: Predicted HLA-I alleles for T-cell epitopes of antigen ESAT-6 of *M. tuberculosis* H37Rv**

**Table 6:** **Predicted HLA- I alleles for T-cell epitopes of antigen CFP-10 of *M. tuberculosis* H37Rv**

| **Start amino acid position** | **Epitope** | **HLA-I allele(s)** |
| --- | --- | --- |
| 3 | EMKTDAATL | HLA-A24,HLA-A*33:02,HLA-A20 Cattle,HLA-A2.1,HLA-B14,HLA-B*27:05,HLA-B*35:01,HLA-B*51:01,HLA-B*53:01,HLA-B*54:01,HLA-B*51,HLA-B7,HLA-B*07:02,HLA-B8,HLA-Cw*04:01,HLA-Cw*06:02,MHC-Kd,MHC-Ld |
| 31 | QVESTAGSL | HLA-A*02:05,HLA-A24,HLA-A68.1,HLA-A20 Cattle,HLA-A2.1,HLA-B*27:05,HLA-B*38:01,HLA-B*39:01,HLA-B*39:02,HLA-B*51:01,HLA-B*53:01,HLA-B*51,HLA-B7,HLA-B*07:02,HLA-Cw*03:01,HLA-Cw*04:01,HLA-Cw*06:02,MHC-Kd,MHC-Ld |
| 41 | GQWRGAAGT | HLA-A*02:01,HLA-A*02:05,HLA-A2.1,HLA-B*27:02,HLA-B*27:05,HLA-B*52:01,HLA-B62,HLA-B*07:02,MHC-Ld |
| 42 | QWRGAAGTA | HLA-B*53:01,HLA-B*54:01,HLA-B*51,HLA-Cw*04:01,MHC-Kd |
| 45 | GAAGTAAQA | HLA-B40,HLA-B*51:01,HLA-B*51:02,HLA-B*51:03,HLA-B*54:01, HLA-B61,HLA-B*07:02 |
| 68 | GTAAQAAVV | HLA-A68.1,HLA-A20 Cattle,HLA-A2.1,HLA-B*51:01,HLA-B*51: 02,HLA-B*51:03,HLA-B*52:01,HLA-B*53:01,HLA-B*54:01,HLA-B*51,HLA-B*58:01, HLA-B61,MHC-Kd,MHC-Ld |
| 49 | TAAQAAVVR | HLA-A*33:02,HLA-A68.1,HLA-A20 Cattle,HLA-B*27:05 |

**Table 7:** **Predicted HLA-II alleles for T-cell epitopes of antigen ESAT-6 of *M. tuberculosis* H37Rv**

| **Start amino acid**  **position** | **Epitope** | **HLA II- allele(s)** |
| --- | --- | --- |
| 5 | WNFAGIEAA | HLA-DRB1*03:05,HLA-DRB1*04:01,HLA-DRB1*04:26,HLA-DRB1*11:14,HLA-DRB1*13:23, |
| 7 | FAGIEAAAS | HLA-DRB1*01:01,HLA-DRB1*04:01,HLA-DRB1*04:05,HLA-DRB1*0408,HLA-DRB1*0426,HLA-DRB1*08:02,HLA-DRB1*11:01,HLA-DRB1*11:28,HLA-DRB1*13:05,HLA-DRB1*13:07, |
| 10 | IEAAASAIQ | HLA-DRB1*04:10, |
| 17 | IQGNVTSIH | HLA-DRB1*03:06,HLA-DRB1*03:08,HLA-DRB1*03:11,HLA-DRB1*04:01,HLA-DRB1*04:02,HLA-DRB1*04:04,HLA-DRB1*04:05,HLA-DRB1*04:08,DRB1*04:10,HLA-DRB1*04:21,HLA-DRB1*04:23,HLA-DRB1*04:26,HLA-DRB1*11:07,HLA-DRB1*13:04, |
| 21 | VTSIHSLLD | HLA-DRB1*04:05,HLA-DRB1*04:10,HLA-DRB1*13:21, |
| 42 | WGGSGSEAY | HLA-DRB1*04:01,HLA-DRB1*04:21,HLA-DRB1*04:26,HLA-DRB1*07:01,HLA-DRB1*07:03, |
| 50 | YQGVQQKWD | HLA-DRB1*13:21, |
| 68 | LQNLARTIS | HLA-DRB1*03:01,HLA-DRB1*03:05,HLA-DRB1*03:06,HLA-DRB1*03:08,HLA-DRB1*03:09,HLA-DRB1*03:11,HLA-DRB1*08:04,HLA-DRB1*08:13,HLA-DRB1*11:01,HLA-DRB1*11:02,HLA-DRB1*11:04,HLA-DRB1*11:06,HLA-DRB1*11:07,HLA-DRB1*11:14,HLA-DRB1*11:20,HLA-DRB1*11:21,HLA-DRB1*11:28,HLA-DRB1*13:01,HLA-DRB1*13:02,HLA-DRB1*13:04,HLA-DRB1*13:05,HLA-DRB1*13:07,HLA-DRB1*13:11,HLA-DRB1*13:21,HLA-DRB1*13:22,HLA-DRB1*13:23,HLA-DRB1*13:27,HLA-DRB1*13:28,HLA-DRB1*15:06 |

**Table 8:** **Predicted HLA-II alleles for T-cell epitopes of antigen CFP-10 of *M. tuberculosis* H37Rv**

| **Start amino acid**  **position** | **Epitope** | **HLA-II allele(s)** |
| --- | --- | --- |
| 3 | MKTDAATLA | HLA-DRB1*03:01,HLA-DRB1*03:05,HLA-DRB1*03:06,HLA-DRB1*03:07,HLA-DRB1*03:08,HLA-DRB1*03:11,HLA-DRB1*11:07, |
| 17 | FERISGDLK | HLA-DRB5*01:01,HLA-DRB5*01:05 |
| 20 | ISGDLKTQI | HLA-DRB1*03:01,HLA-DRB1*03:05,HLA-DRB1*03:06,HLA-DRB1*03:07,HLA-DRB1*03:08,HLA-DRB1*03:09,HLA-DRB1*03:11,HLA-DRB1*11:07, |
| 42 | WRGAAGTAA | HLA-DRB1*01:01,HLA-DRB1*01:02,HLA-DRB1*04:08,HLA-DRB1*08:13,HLA-DRB5*01:01,HLA-DRB5*01:05 |
| 54 | VVRFQEAAN | HLA-DRB1*04:08,HLA-DRB1*08:06,HLA-DRB1*13:04, |
| 55 | VRFQEAANK | HLA-DRB1*01:01,HLA-DRB1*01:02,HLA-DRB1*03:05,HLA-DRB1*03:06,HLA-DRB1*03:07,HLA-DRB1*03:08,HLA-DRB1*03:11,HLA-DRB1*04:01,HLA-DRB1*04:02,HLA-DRB1*04:04,HLA-DRB1*04:21,HLA-DRB1*04:23,HLA-DRB1*04:26,HLA-DRB1*08:04,HLA-DRB1*11:01,HLA-DRB1*11:02,HLA-DRB1*11:04,HLA-DRB1*11:06,HLA-DRB1*11:07,HLA-DRB1*11:14,HLA-DRB1*11:20,HLA-DRB1*11:21,HLA-DRB1*13:01,HLA-DRB1*13:02,HLA-DRB1*13:04,HLA-DRB1*13:07,HLA-DRB1*13:11,HLA-DRB1*13:22,HLA-DRB1*13:23,HLA-DRB1*13:27,HLA-DRB1*13:28,HLA-DRB5*01:01,HLA-DRB5*01:05 |
| 57 | FQEAANKQK | HLA-DRB5*01:01,HLA-DRB5*01:05 |
| 75 | IRQAGVQYS | HLA-DRB1*01:02,HLA-DRB1*03:01,HLA-DRB1*03:05,HLA-DRB1*03:06,HLA-DRB1*03:07,HLA-DRB1*03:08,HLA-DRB1*03:09,HLA-DRB1*03:11,HLA-DRB1*04:01,HLA-DRB1*04:02,HLA-DRB1*04:04,HLA-DRB1*04:05,HLA-DRB1*04:08,HLA-DRB1*04:10,HLA-DRB1*04:10, HLA-DRB1*04:21,HLA-DRB1*04:23,HLA-DRB1*04:26,HLA-DRB1*08:01,HLA-DRB1*08:02,HLA-DRB1*08:04,HLA-DRB1*08:06,HLA-DRB1*08:13,HLA-DRB1*11:01,HLA-DRB1*11:02,HLA-DRB1*11:04,HLA-DRB1*11:06,HLA-DRB1*11:07,HLA-DRB1*11:14,HLA-DRB1*11:20,HLA-DRB1*11:21,HLA-DRB1*11:28,HLA-DRB1*13:01,HLA-DRB1*13:02,HLA-DRB1*13:04,HLA-DRB1*13:05HLA-,DRB1*13:07,HLA-DRB1*13:11,HLA-DRB1*13:21,HLA-DRB1*13:22,HLA-DRB1*13:23,HLA-DRB1*13:27,HLA-DRB1*13:28 |
